# COPASutils: An R Package for Reading, Processing, and Visualizing Data from COPAS Large-Particle Flow Cytometers

**DOI:** 10.1371/journal.pone.0111090

**Published:** 2014-10-20

**Authors:** Tyler C. Shimko, Erik C. Andersen

**Affiliations:** Department of Molecular Biosciences, Northwestern University, Evanston, Illinois, United States of America; Inserm U869, France

## Abstract

The R package COPASutils provides a logical workflow for the reading, processing, and visualization of data obtained from the Union Biometrica Complex Object Parametric Analyzer and Sorter (COPAS) or the BioSorter large-particle flow cytometers. Data obtained from these powerful experimental platforms can be unwieldy, leading to difficulties in the ability to process and visualize the data using existing tools. Researchers studying small organisms, such as *Caenorhabditis elegans*, *Anopheles gambiae*, and *Danio rerio*, and using these devices will benefit from this streamlined and extensible R package. COPASutils offers a powerful suite of functions for the rapid processing and analysis of large high-throughput screening data sets.

## Introduction

High-throughput screening is an increasingly important task in many fields of biology [1]. The Complex Object Parametric Analyzer and Sorter (COPAS) platform and the BioSorter device from Union Biometrica allow for rapid and automated collection of flow data, including object length, optical density, and three fluorescence channels, from a large variety of organisms. Comprising five distinct machines, these devices can be used to analyze and sort organisms ranging in size from 10–1500 microns, including *Caenorhabditis elegans* [2], *Anopheles gambiae* [3], and *Danio rerio* [4]. Data are output in a flat, tab-delimited text file format, which is machine-readable. With the addition of an optional ReFLx module or LP sampler, samples are collected from a standard 96-well microtiter plate, allowing for well-by-well analysis. Although tab-delimited text files are read and understood by machines with existing software tools, they do not represent the best human-interpretable way to analyze and visualize 96-well data.

A large number of software environments can be used for analysis of data from these large particle measurement devices, including R, SAS, and MATLAB. The R environment offers several distinct advantages over other programming environments, including free and open-source software packages along with a vibrant community of scientists developing novel software. Two existing packages could be used to analyze COPAS data, but they are limited to the expensive MATLAB environment [5] or specifically written for cell culture RNAi experiments [6]. R is the standard for statistical computing and the fastest growing environment for analysis of large data sets in computer science and biology.

We developed COPASutils, a novel software package for the R statistical programming environment to assist in the reading, processing, analysis, and visualization of data from 96-well plates, specifically data from the different COPAS platforms. COPASutils leverages the most recently developed R tools to rapidly read, summarize, and clean data to present elegant and adaptable visualizations of both summary and distribution statistics in a complete, compact, and intuitive pipeline structure. It represents a significant improvement over existing software packages by leveraging the R environment and research community to analyze data specifically generated using the powerful large-particle flow cytometers from Union Biometrica.

### For users new to R

We recommend searching online resources for introductions to R. Tutorials for the interface, data handling, and scripting are the most useful introductions. The source code of the COPASutils package is available on the Comprehensive R Archive Network repository and can be installed by typing the following command in R: install.packages(“COPASutils”). For a tutorial on the usage of COPASutils and the available functions, please see the vignette at http://andersenlab.org/files/COPASutilsVignette.html. This package is open-source. For updates and to submit comments, please go to https://github.com/AndersenLab/COPASutils.

### Design and Implementation

**Figure 1:**
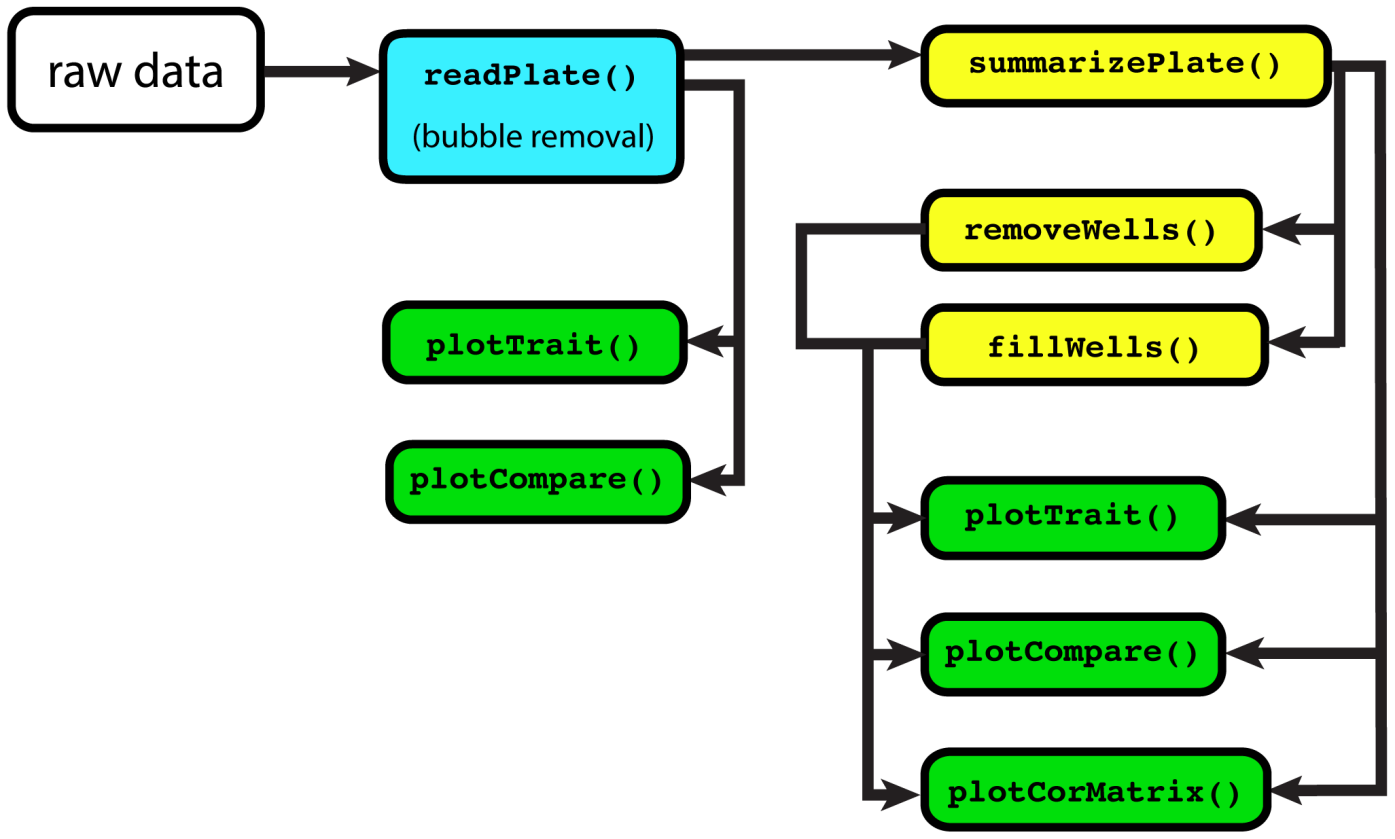
COPASutils workflow map. Suggested workflow for COPAS data using the COPASutils package. Reading steps are shown in blue, processing steps are shown in yellow, and plotting steps are shown in green.

COPASutils is designed to be simple to make it addressable to users familiar with the R environment. The functions included in the package have been constructed in such a way as to adapt to the data entered as input, so that users are not overwhelmed by options when attempting to use the functions. In the implementation of the COPASutils package, speed and extensibility are paramount. Data summarization functions are built upon the dplyr package [7] to minimize computational time and load. In addition, all plotting functions are built upon the popular ggplot2 visualization package [8] and, as such, resultant plots can be further modified to fit user specifications using standard ggplot2 grammar and functions. The complete source code and binaries for COPASutils are available from the Comprehensive R Archive Network (http://cran.r-project.org/web/packages/COPASutils). Because the entirety of the COPASutils package is written in the R language, this package is easily portable across platforms as well as completely free to utilize and modify. Overall, COPASutils presents a branched linear workflow for the analysis of COPAS data (Figure 1) that is simple for novice users of R as well as extensible for experienced users and users with specific experimental designs.

### Data import and bubble detection

Data from a COPAS machine are output to tab-delimited text files. Each line of the text file represents the observation of one object that passed through the flow cell. However, the COPAS software cannot differentiate objects of interest from bubbles or particulate contamination. This issue is common for projects that require liquid handling with the bypass of the bubble trap hardware or for experiments where a chemical precipitate is present. Consequently, we have designed a function to read in the data that can eliminate many of these false-positive observations. The function required to read in the data can optionally set minimum and maximum cutoffs for the size and optical density of the objects, allowing the user to exclude data objects beyond the size parameters of the organism being measured. This function works for data obtained from any flow cytometer with the ability to sample from 96-well plates, including the COPAS and BioSorter platforms.

Additionally, the function can optionally employ a support vector machine (SVM) [9] to probabilistically determine if an object recorded by the COPAS software is an undesirable bubble or a true object to be measured. This support vector machine must be trained on each COPAS or BioSorter device because internal parameters vary by device. To train the SVM, run two 96-well microtiter plates through your device. The first plate should contain only objects of interest with the bubble trap engaged, and the second plate should contain no objects of interest, less liquid per well, and have the bubble trap disengaged. These plates will generate object and bubble data, respectively. A variety of R packages (like [9]) can be used to generate an SVM for use with the readPlate function. With the combination of particulate contamination reduction and bubble detection and removal, the COPASutils package represents a powerful method of data filtering that greatly reduces the number of false objects analyzed.

The function used to read in plate data will also normalize the optical density and fluorescence values measured for every object. The device outputs integrated measures of each of these parameters. Therefore, larger objects always have increased optical density or fluorescence values. To better represent the optical density or fluorescence of individual objects, we divide each parameter by the length (time of flight) for every object recorded. This normalization allows the researcher to determine if objects of different size also have different optical density and fluorescence.

### Summarization, well removal, and data filling

After the data are read in, it is often necessary to summarize the data by well in order understand patterns by strain, treatment, or replicate. Therefore, we have included a function to aid in the summarization of the data by well. The summarization function calculates not only means and variances of all distributions of measured parameters but also quantiles along with minimum and maximum values. Additionally, this function can also summarize these statistical measures for log-transformed parameters. These values can describe the center, spread, and tails of the distributions for each feature, separated by each well of a microtiter plate.

The package also contains tools for further processing of the data after it has been read into R and summarized. COPAS or BioSorter machines with either a ReFLx module or LP sampler are capable of measuring object characteristics for every object from each well in a 96-well plate. However, some experimental designs do not require the use of all 96 wells. For this reason, the package includes a function for the removal of specific wells from the data set, either by setting all trait data from those wells to NA or by dropping those entries entirely. The function accepts a character vector of wells to be removed as input, allowing for well removal to be scripted and automated based on parameters of the summarized data.

Moreover, if wells are unused in a particular experimental design, the COPAS or BioSorter machines might fail to record any observations from the empty wells. This scenario may be problematic for the downstream steps of processing and visualizing the output data. To compensate for unused wells in the summarized data, a function has been constructed that will automatically detect missing wells and add the wells to the summarized data. The wells will be included in the summarized data with NAs for all of the population statistics, making further processing and visualization consistent.

### Plotting and cross-plate comparisons

**Figure 2:**
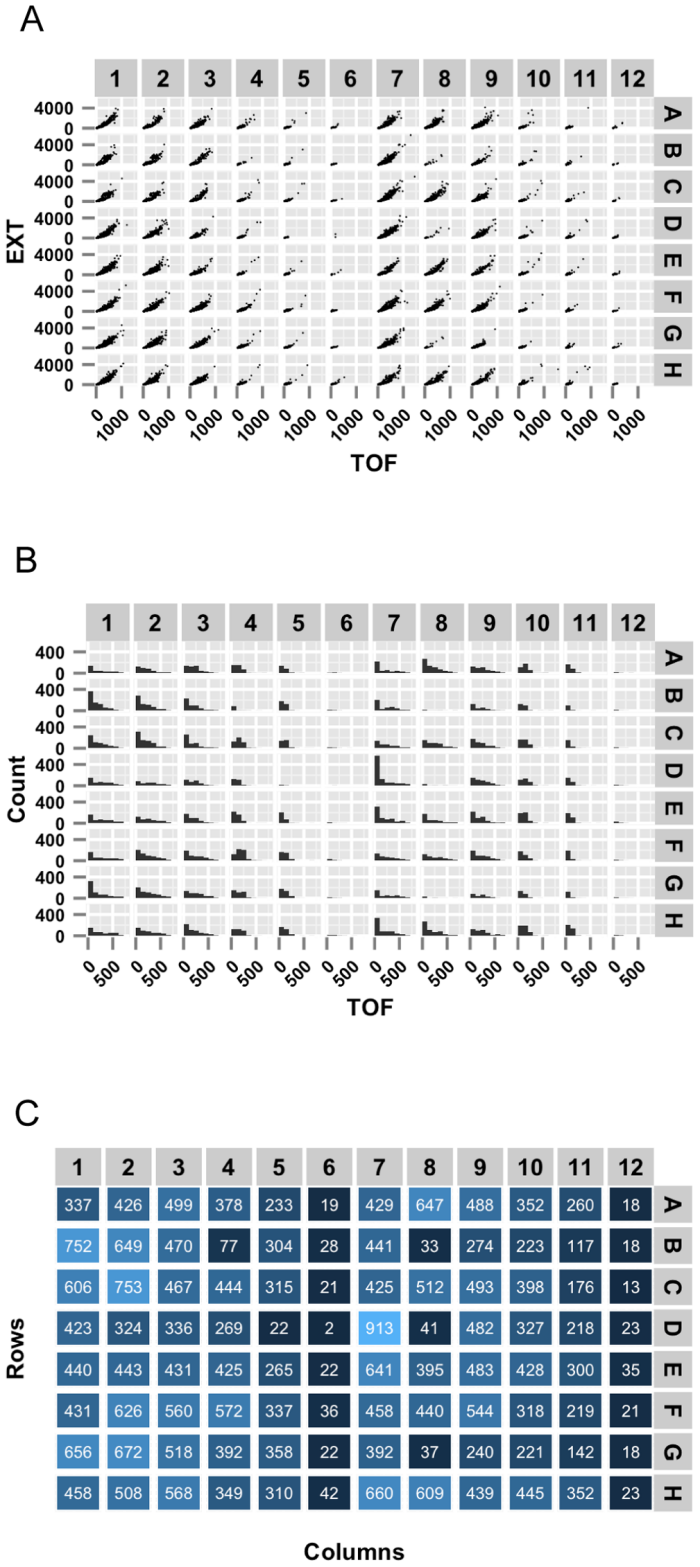
Example trait plots. Three possible plots made using the plotTrait function. (A) Well-wise scatter plots of the time-of-flight (TOF, a measure of length) values plotted against the extinction (EXT, a measure of optical density) values from raw data. (B) Histograms of the TOF values from raw data. (C) Heatmap of the population size from the summarized data (variable n) of each well. All data from this figure are included with the COPASutils package and deposited at https://github.com/AndersenLab/COPASutils-data and http://andersenlab.org/Research/Software/.

Because summarization might reduce the amount of data analyzed by the user, it is often best to visualize both the distribution of the original data and the values of the summary statistics to recognize patterns and identify potential errors. Included in the package are several plotting functions that make the visualization steps of the analysis simple and beautiful. The first of these plotting functions generates scatter plots and histograms for traits in a raw data frame (Figure 2a and 2b) or heatmap plots for traits in a summarized data frame (Figure 2c). This plotting function is particularly advantageous as it allows users to explore correlations between wells or between traits within the same well.

**Figure 3:**
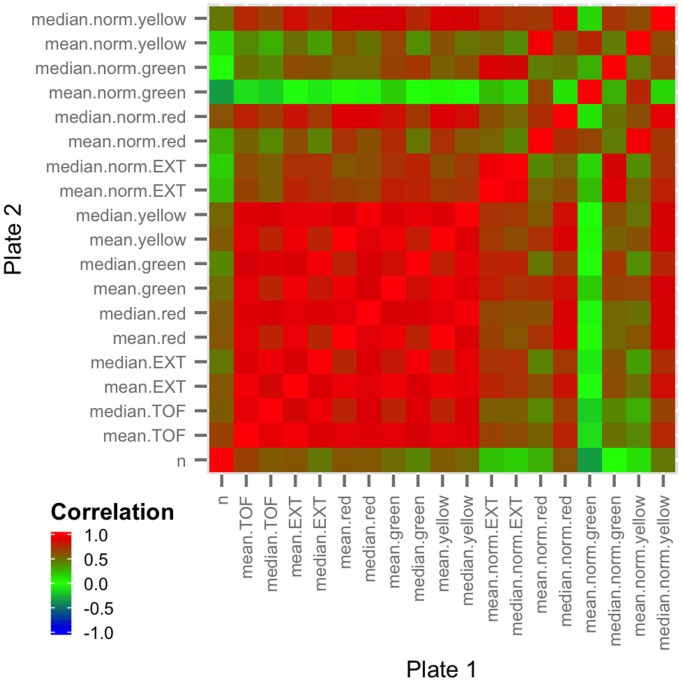
Example correlation matrix plot. A correlation matrix is plotted for all traits within a single plate using the plotCorMatrix function. Correlation matrix plots can be generated within a single plate (as shown) or between two plates. Positively correlated traits are shown in red, uncorrelated traits are shown in green, and negatively correlated traits are shown in blue. In this plot, all traits related to the mean and median values of optical properties of the objects (EXT, red, yellow, green) appear highly correlated, indicating that it may not be useful to include all such traits in further analysis.

Correlations can also be explored on a much larger scale within the summarized data. We have included a separate function for the plotting of a correlation matrix between all traits in a summarized data frame either within a plate or between plates. Employing this function, users can determine which of the observed traits correlate with one another (Figure 3). For example, a user can recognize that the mean length of an organism is negatively correlated with population size, indicating that there could be competition for resources such as nutrients or oxygen. This type of relationship is experimentally relevant and immediately identifiable using a correlation matrix between traits.

**Figure 4:**
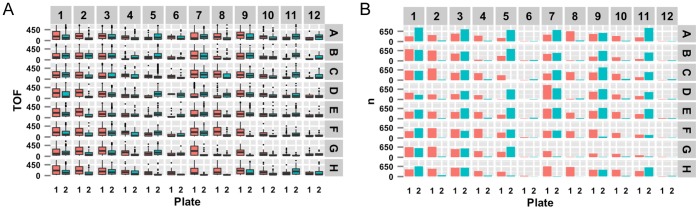
Example comparison plots. Two possible plots made using the plotCompare function. (A) Cross-plate comparisons, from raw data, of the distribution of the time of flight values within each well. (B) Comparison of population sizes, from summarized data, between two plates. All data from this figure are included with the COPASutils package and deposited at https://github.com/AndersenLab/COPASutils-data and http://andersenlab.org/Research/Software/.

Additionally, we have included a function to generate plots that allow the user to compare distributions (Figure 4a) or summary statistics across plates (Figure 4b). Distributions of raw data are represented as box and whisker figures in a boxplot. Similarly, summarized data are represented as bars in a bar plot. All of the individual plots are drawn in the position of their respective wells in a standard 96-well format. In this manner, data in the same well can be directly compared across plates. Even though plate-well series plots have previously been considered for this type of cross-plate comparison [10], the well-by-well plots presented in the COPASutils package represent a more straightforward representation of data which do not necessarily display any trend with increasing well number. Therefore, the plotting functions included in the COPASutils package are expected to have broader applicability than others currently available.

### Dose response experiments

**Figure 5:**
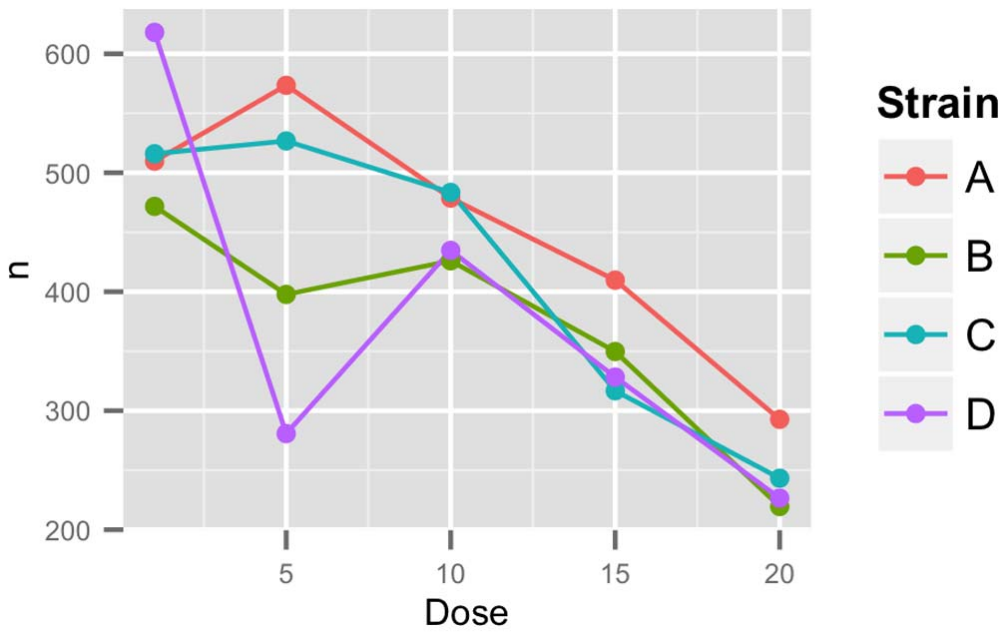
Example dose response plot. A dose response curve is plotted for four different strains in five different conditions using the plotDR function. Each point represents a single observation. Lines represent overall change in population size between doses. Dose response curves can be plotted for any trait present in the summarized data frame.

COPAS machines are particularly well equipped to measure dose-dependent responses after exposure of animals or cells to increasing concentrations of drugs, RNAi, or other environmental perturbations. Because of the widespread utilization of the large-particle sorting devices for these experiments [11] functions have been included that allow the plotting of dose-response data (Figure 5). These functions require additional input from the user in the form of two 96-element vectors, one for the strains present in the plate, which is passed to the summarization function, and one for the dosages used, passed to the plotting function. The user then specifies the trait to be examined and a plot will be generated that summarizes the trait by strain and plots the mean of the selected trait with increasing dosage. A second function is also included that will return a list of plots for all traits present in the dataset, which allows for the plotting and analysis to be scripted.

### Edge-effect detection

For long-running experiments, one can observe effects caused by the position of the well within the microtiter plate. These effects, caused by uneven oxygenation or evaporation, can obfuscate measurements of populations within wells of the plate. For instance, the outermost wells of a plate are most likely to experience the greatest evaporation, which might affect the outcome of a particular experiment. In order to test for these “edge-effects,” we have included a function that will split the plate into two populations, edge wells and center wells, and return the *p*-value(s) for a two-sided Wilcoxon Rank-Sum test for either a user-defined trait or for all traits. By utilizing this function, users can recognize plates that were affected by well position effects and remove those data from any further analyses. Users implementing the COPASutils package can ensure that their data are as clean and consistent as possible.

## Conclusions

The COPASutils package provides simple reading, processing, and plotting tools for data obtained from the powerful COPAS platforms in the R statistical programming environment. The package presents an organized workflow for the management of COPAS data. By leveraging existing R tools, COPASutils offers rapid summarization and modular visualization design that is extensible to many unique projects. In addition, the package includes tools that offer compact and understandable visualizations for comparing data across wells, plates, or functions for early-stage detection of anomalous data. COPASutils is a simple, extensible, and reproducible open-source analysis pipeline for COPAS data.
